# Some Details as to Aseptic Operation

**Published:** 1900-03-03

**Authors:** 


### SOME DETAILS AS TO ASEPTIC OPERATION.

In speaking at the Leamington Medical Society on the organisation of aseptic operation Mr. Lockwood described the measures which he had found to be most effectual for producing asepsis. For the disinfection of the skin of the patient steps need not be taken overnight. All that is necessary can be done an hour or so before operation, and in the case of a lady or child may even be deferred until the patient is under the anæsthetic. The steps are as follows: (1) The skin is, if necessary, shaved; (2) it is thoroughly washed with soap and water; (3) as much as possible of the remaining fat is extracted with ether, or turpentine, or benzine, turpentine being probably the best; and (4) the skin is thoroughly saturated for not less than two minutes with a solution of biniodide of mercury (rendered soluble by the addition of potassium iodide) in methylated spirit (1 in 500). For the disinfection of the hands after the nails have been cut short and all outside dirt and grease removed, the above solution of biniodide of mercury and methylated spirit is employed. Not less than half a pint of this fluid should be placed in a capacious sterilised bowl, and the disinfection should in many cases be made to extend to the wrists and forearms. Should this process cause roughness of the skin during inclement weather a mixture of glycerine and eau de Cologne applied at night brings about a speedy cure. Instruments are boiled for 15 to 20 minutes in a solution of sodium carbonate (one drachm to the pint), and at the time of the operation lie in carbolic lotion (1 in 60). For sutures and ligatures fishing gut and one or two sizes of twisted silk are used. These may be sterilised by boiling for 20 minutes in water, and then placing them in a jar of 1 in 20 carbolic lotion for conveyance to the operation. Silk should not be boiled in soda, or it will be rendered brittle. Towels are sterilised by boiling for half an hour in a boiler or saucepan, after which they are transferred to 1 in 40 carbolic lotion. Sponges are beaten, and the shell and coral dissolved out with dilute hydrochloric acid, and then treated with washing soda; and, lastly, the sponge is disinfected and bleached by being submerged in a 1 in 5 solution of sulphurous acid in water. During the operation the sponges and the hands of the operator are constantly washed with biniodide of mercury lotion, 1 in 2,000. As small a number of sponges as possible is employed; often only one. Four round and one flat sponge should suffice for an abdominal operation.

